# Differences in Plasma Exposure of Cannabidiol and Cannabidiolic Acid Following Oral Administration to Horses

**DOI:** 10.1111/jvp.70027

**Published:** 2025-09-28

**Authors:** Carl Ekstrand, Peter Michanek, Elin Hernlund, Ronette Gehring, Kristin Spjut, Matilda Salomonsson

**Affiliations:** ^1^ Department of Animal Biosciences, Division of Pharmacology and Toxicology Swedish University of Agricultural Sciences Uppsala Sweden; ^2^ Department of Population Health Sciences, Division of Veterinary and Comparative Pharmacology Utrecht University Utrecht the Netherlands; ^3^ Department of Chemistry, Environment and Feed Hygiene Swedish Veterinary Agency (SVA) Uppsala Sweden; ^4^ Department of Medicinal Chemistry, Analytical Pharmaceutical Chemistry Uppsala University Uppsala Sweden

**Keywords:** anti‐doping, cannabinoids, equines, pharmacokinetics

## Abstract

There has been a growing interest in the use of cannabinoids in horses in recent years. Several studies have reported on the pharmacokinetics of cannabidiol (CBD) in horses. However, cannabidiolic acid (CBDA) has received less attention, despite limited evidence suggesting clinically beneficial effects in other species. Horses were administered 3 mg/kg of CBD, 3 mg/kg of CBDA, and a placebo per os in a crossover design, with a one‐week washout period between treatments. Plasma and urine samples were collected and analyzed using ultra high‐performance liquid chromatography coupled to tandem mass spectrometric. Observed CBDA plasma concentrations were up to 67 times higher, and the CBDA area under the plasma concentration‐time curve was up to 36 times larger than those of CBD. Median terminal half‐lives in plasma were 7.8 h for CBD and 5.3 h for CBDA. Both compounds were detectable in plasma for up to 72 h. In urine, CBD and CBDA were detectable for 168 and 72 h, respectively. The results suggest greater intestinal uptake or lower first‐pass metabolism/clearance of CBDA compared to CBD. Given the poor oral bioavailability of CBD in horses, CBDA may hold greater clinical relevance. Further studies are needed to elucidate the pharmacokinetics and pharmacodynamics of CBDA in horses.

## 1 Introduction

Approximately 70 different cannabinoids have been isolated from hemp ( *Cannabis sativa* , *Cannabis indica* , and *Cannabis ruderalis*) (Karst et al. 2010). As early as the 1960s, Δ^9^‐tetrahydrocannabinol (THC) and cannabidiol (CBD) were isolated and synthesized (Gaoni and Mechoulam 1964). Both THC and CBD are active substances in pharmaceutical products used for pain relief and muscle relaxation in humans. Unlike THC, CBD has been proposed to have minimal psychoactive effects.

In veterinary medicine, the pharmacokinetics and clinical efficacy of CBD were first reported in dogs and cats (Samara et al. 1988; Gamble et al. 2018; Verrico et al. 2020; Deabold et al. 2019). More recently, interest has grown for its use in horses. The pharmacokinetics of CBD have been described following intravenous administration (Sánchez de Medina et al. 2023; Turner et al. 2022), and systemic exposure after oral administration—using various formulations and either single or multiple dosing regimens—has also been published (Leise et al. 2023; Ryan et al. 2021; St Blanc et al. 2022; Williams et al. 2022; Yocom et al. 2022; Eichler et al. 2023). Additionally, case reports suggest potential benefits of CBD in managing stereotypic behavior and mechanical allodynia in horses (Cunha et al. 2023; Ellis and Contino 2021), while smaller clinical studies have reported analgesic and anti‐inflammatory effects after transmucosal administration of CBD or a combination of CBD and cannabigerol (Aragona et al. 2024; Interlandi et al. 2024).

Cannabidiolic acid (CBDA) is another cannabinoid, structurally similar to CBD. CBDA is an aromatic carboxylic acid that is readily converted to CBD through decarboxylation when exposed to light, air, or heat (Turner et al. 1980; Citti et al. 2018). Preclinical and clinical studies, including in vitro assays and rodent models, suggest that CBDA has anti‐inflammatory, anti‐nociceptive, and anti‐convulsing properties, largely without psychotropic effects (Takeda et al. 2008; Vigli et al. 2021; Anderson et al. 2019; Rock et al. 2018). Pharmacokinetic data on CBDA have been reported in dogs, horses, and parrots (Wakshlag et al. 2020; Sosa‐Higareda et al. 2023; Wang et al. 2025), and CBDA has been proposed for anti‐allergy therapy in dogs due to its ability to mitigate cytokine production in vitro in cells from dogs with atopic dermatitis (Chiocchetti, De Silva, et al. 2022; Chiocchetti, Salamanca, et al. 2022). Co‐administration of CBDA and CBD has also shown effects on pruritus and seizures in dogs (Loewinger et al. 2022; Garcia et al. 2022) In horses, a study on 24 lame individuals reported analgesic effects from transmucosal CBDA (Aragona et al. 2024). In contrast, a clinical study in dogs did not demonstrate anti‐nociceptive effects with co‐administration of CBD and CBDA (Klatzkow et al. 2022).

In horses, the oral bioavailability for CBD is low, < 15% (Turner et al. 2022; Sánchez de Medina et al. 2023), which may limit the achievement of therapeutic plasma concentrations after oral administration. In contrast, the plasma exposure of CBDA has been shown to be higher than that of CBD following oral administration in dogs and parrots (Wakshlag et al. 2020) (Sosa‐Higareda et al. 2023). Similarly, administration of hemp‐based oil containing multiple cannabinoids, such as CBD, CBDA, and THC, in horses resulted in higher plasma concentrations of CBDA compared to CBD (Thomson et al. 2024).

The primary objective of this study was to investigate and further compare the plasma and urine concentrations of CBD and CBDA following a single oral administration in horses, using products containing only one cannabinoid. A secondary aim was to assess potential side effects associated with this administration.

## 2 Material and Methods

### 2.1 Drugs and Chemicals

A commercially available CBD‐oil (Bedrolite 100 mg/mL, Cannabiszorg, Breukelen, the Netherlands) was used in this study. The CBD preparation was produced according to Good Manufacturing Practice (GMP) and, according to the manufacturer, contained traces of other cannabinoids (CBDA 0.16%, THC 0.45%, and cannabichromene 0.45%). The CBDA preparation (100 mg/mL in almond oil) was compounded by Clinical Cannabis Care (Breukelen, the Netherlands) using CBDA raw material supplied by Purisys (Athens, GA, USA).

### 2.2 Animals and Preparation

Eight horses (five mares and three geldings) were included in the study. One horse participated in a pilot study, and seven were enrolled in the main experiment. The horses ranged in age from 2 to 22 years and weighed between 450 and 585 kg. All horses were considered chronically lame based on history and repeated objective motion analyses but were otherwise clinically healthy.

Following oral administration of CBD, CBDA, or placebo, the horses were housed in individual boxes for 36 h. After this period, they were allowed access to paddocks or pasture during daytime and returned to their box at night. The horses were fed haylage and concentrate consistent with their regular diet throughout the study. Water was available *ad libitum*.

Prior to administration, the hair over one of the jugular veins was clipped, and the skin was pre‐treated with a topical anesthetic cream (EMLA; 25 mg/g prilocain + 25 mg/g lidocaine, Aspen Nordic, Ballerup, Denmark). A catheter (MILA, 14G [2.1 × 130 mm] Florence, Kentucky, USA) was then placed.

The study protocol was approved by the Animal Ethics Committee, Uppsala, Sweden (5.8.18‐02809/2020).

### 2.3 Experimental Design

The trial followed a three‐treatment, randomized, blinded crossover design with a minimum one‐week washout period between treatments. Fed horses received either CBD, CBDA, or placebo (saline), which was administered intragastrically via an infusion extension line (Heidelberg type) inserted into a gastric tube. After each administration, the line was flushed with 60 mL of rapeseed oil followed by 120 mL of water. A new extension line was used for each treatment.

Blood samples were collected into evacuated tubes containing either EDTA 2K (for plasma) or clot activator (for serum). All samples were placed immediately on ice after collection and centrifuged within 1 h at 1800 *g* for 10 min. The supernatant was transferred to new tubes and stored at −80°C pending analysis.

For the pilot study (Horse #1): CBD and CBDA were administered at a dose of 1 mg/kg followed by 2 mg/kg at 12 h. Blood was collected pre‐administration (0 h) and at 0.33, 0.67, 1, 1.5, 2, 3, 4, 6, 8, 12, 12.33, 12.67, 13, 13.5, 14, 15, 16, 18, 20, 24, 30, 36, 42, 48, 72, 120, and 168 h after the first administration. Blood samples for hematology and biochemistry were drawn pre‐administration and at 168 h. Urine samples were collected pre‐administration (0 h) and at 4, 12, 16, 24, 36, 48, 72, 120, and 168 h post first administration.

For the main experiment (Horses #2–#8): A single dose of 3 mg/kg of either CBD or CBDA was administered at 0 h, corresponding to 9.54 and 8.37 μmol/kg CBD and CBDA, respectively. Blood samples were collected pre‐administration (0 h) and at 0.33, 0.67, 1, 1.5, 2, 3, 4, 6, 8, 12, 16, 20, 24, 30, 39, 48, 72, 120, and 168 h post‐administration. Blood samples for hematology and biochemistry were collected at 0 and at 168 h. Urine was sampled pre‐administration (0 h) and at 4, 12, 24, 39, 48, 72, 120, and 168 h post‐administration.

### 2.4 CBD and CBDA Analyses

The reference material CBD was purchased from Chiron AS (Trondheim, Norway), CBDA and internal standards CBD‐d9 and CBDA‐d3 were purchased from Cayman Chemical (Ann Arbor, USA). The water was purified with a MilliQ purification system from Merck Life Science AB (Merck KGaA, Darmstadt, Germany). All other chemicals used were of analytical grade or better.

An Acquity UPLC coupled to a Xevo TQ‐Sμ tandem quadrupole mass spectrometer (Waters Corporation, MA, USA) with an electrospray interface was used for the quantification.

The separation was performed using a chromatographic system consisting of a C18 guard column and an Acquity BEH C18 column (2.1 × 50 mm, 1.7 μm) (Waters Corp.) both at 65°C. The mobile phase was delivered as a gradient consisting of (A) methanol and (B) 0.1% formic acid in water. The gradient started at 30% A at 0.50 min, then continued with a linear increase over time to 80% A at 4.00 min, and 95% A at 5.50 min. At 6.80 min the gradient switched to 30% A for equilibration. The flow rate was 0.400 mL/min and the injection volume was 10 μL. The ionization was positive electrospray, and the instrument settings were a capillary voltage of 1.5 kV, a cone voltage of 23 V, a source temperature of 150°C, a desolvation temperature of 500°C, and a desolvation gas flow of 1000 L/h. The analytes were detected as [M + H]^+^ and the analysis mode was Selected Reaction Monitoring (SRM). The SRM transitions used for the quantitative analyses were CBD m/z 315 > 193 (collision energy 20 eV), CBD‐d9 m/z 324 > 202 (collision energy 22 eV), CBDA m/z 359 > 219 (collision energy 30 eV), and CBDA‐d3 m/z 362 > 222 (collision energy 32 eV). The dwell time was 0.025 s, and the results were evaluated using the software TargetLynx (Waters Corp.).

The preparation of plasma samples (including study samples, calibrators, or quality control samples) was carried out in a 96‐well plate according to the following procedure: To each well, 100 μL of plasma was added along with 100 μL of an acetonitrile solution (blank, calibrator, or QC), and 50 μL of an internal standard solution containing 1.5 ng/mL of CBD‐d9. Following this, an additional 300 μL of acetonitrile was added for protein precipitation. The samples were then vortexed for 10 min and centrifuged at 4000 *g* for 10 min.

The supernatants (300 μL) were transferred to a new 96‐well plate and evaporated. The residues were then reconstituted in 100 μL 10 mM ammonium acetate (pH 9.3): acetonitrile (v/v, 75:25). The plate was vortexed for 5 min before the samples (10 μL) were injected into the UHPLC–MS/MS system.

The urine samples were extracted using an Isolute Multimode (300 mg/3 mL) solid phase column (Biotage, Sweden). The sample preparation of urine (study samples, calibrators, or quality control samples) was performed as follows: to a centrifuge tube, 1.00 mL urine, 100 μL acetonitrile solution (blank/calibrator/QC), 100 μL internal standard solution (250 ng/mL CBD‐d9 or 150 ng/mL CBDA‐d3), 1.00 mL potassium phosphate buffer solution (pH 6.05), 30 μL β‐glucuronidase, and 20 μL protease were added. The samples were vortexed for 1 min and left at room temperature for 30 min for hydrolysis under horizontal shaking. The samples were then centrifuged at 3500 *g* for 10 min, and the prepared samples were transferred to a RapidTrace instrument (Biotage, Sweden) for solid phase extraction. The column was equilibrated with methanol, milliQ water, and potassium phosphate buffer solution (pH 6.05) before the samples were loaded. The column was rinsed/washed with potassium phosphate buffer solution (pH 6.05), milliQ water, 1 M acetic acid, and methanol before elution with 5 mL methanol/ethyl acetate/dichloromethane/2‐propanol solution (v/v/v/v, 10:1:1.3:5). The samples were evaporated at 50°C, and the residue was dissolved in 200 μL 10 mM ammonium acetate (pH 9.3): acetonitrile (v/v, 75:25), and 10 μL were injected into the UHPLC–MS/MS system.

For the quantitative analysis, calibration curves with eight calibrators (0.076–20.4 ng/mL CBD in plasma, 0.750–150 ng/mL CBDA in plasma, 0.482–48.2 ng/mL CBD in urine, low range 4.45–49.4 ng/mL CBDA in urine and high range 39.5–988 ng/mL CBDA in urine) were used, and the calibration curves were constructed with the peak area ratio (CBD or CBDA/internal standard) as a function of the CBD or CBDA concentration. The calibration functions were calculated by linear regression with a weighting factor of 1/*x* ^2^ (CBD) and 1/*x* (CBDA).

Four levels of quality control samples were prepared in plasma for CBD 0.1, 0.3, 3.0, and 7.0 ng/mL, and also for CBDA at 1.0, 7.0, 50, and 90 ng/mL.

The quality control samples for CBD in urine were at 0.7, 2.0, 25, and 41 ng/mL, and for CBDA at 4.9, 9.9, 25, 40, 45, 2, and 790 ng/mL. Two replicates of each calibrator and six replicates at each QC concentration level were analysed, and the analysis was repeated on three different days.

A method validation (evaluating linearity, limit of quantification (LOQ), precision, and accuracy) was conducted with the bioanalytical method validation guidelines established by the European Medicines Agency (EMA) as a guide, and all results were within the accepted criteria. The linearity was evaluated and the *R* ^2^ values obtained were ≥ 0.99 for both compounds and all days. The LOQ in plasma was determined to be 0.10 ng/mL for CBD and 1.0 ng/mL for CBDA, whereas the LOQ in urine was determined to be 0.74 ng/mL for CBD and 4.9 ng/mL for CBDA. The intra‐ and inter‐day precision (RSD %) in plasma for CBD was in the range of 1.8%–12% and for CBDA in the range of 1.8%–13%. In the urine, the precision varied from 2.2% to 15% for CBD (9.4%–19% at LOQ) and from 0.4% to 3.1% for CBDA. The accuracy in plasma for CBD was between 100% and 110% (100%–119% at LOQ), and for CBDA, it was between 87% and 115%. In urine, the accuracy for CBD was in the range of 86%–111% and for CBDA in the range of 96%–104% (113%–117% at LOQ).

### 2.5 Hematology and Biochemistry Analyses

Blood cell counts were performed using ProCyte Dx (IDEXX Laboratories Inc., Westbrook, Maine, USA). The variables analysed were: red blood cell count, hematocrit, hemoglobin, mean corpuscular volume, mean corpuscular hemoglobin, mean corpuscular hemoglobin concentration, red blood cell distribution width, total white blood cell count, neutrophil count, lymphocyte count, monocyte count, eosinophil count, basophil count, and platelet count.

Biochemical analysis was performed using Architect c4000 (Abbott Laboratories, Lake Forest, Illinois, USA). The variables analysed were: albumin, alkaline phosphatase (ALP), aspartate aminotransferase (ASAT), creatine kinase (CK), bile acids, gamma‐glutamyl transferase (GGT), glutamate dehydrogenase (GLDH), glucose, potassium, sodium, creatinine, serum amyloid A (SAA), total protein, triglycerides, and blood urea nitrogen (BUN).

### 2.6 Pharmacokinetic Analyses

For initial data exploration and to provide input to the population PK analysis, data was subjected to non‐compartmental analyses (NCA) using PKanalix 2024R1 (Lixoft SAS, A Simulations Plus company). The analyses were performed separately for CBD and CBDA in both plasma and urine using a linear up and log down integral method. A minimum of three observations were used for estimation of the slope of the terminal phase (elimination rate constant [*k*]). The parameters *k* and area under the curve extrapolated to infinity (AUC_0–ꚙ_). Maximal observed concentration (*C*_max_), and terminal half‐life (*t*_*1/2z*_) were NCA outputs.

Pharmacokinetic compartment modeling was performed using Monolix 2024R1 (Lixoft, Antony, France) to fit a non‐linear mixed effects (NLME) model to the experimental data. Model evaluation was based on graphical inspection of diagnostic plots (including individual fits, observed vs. predicted concentration, weighted residuals vs. time, and weighted residuals vs. concentration) as well as the visual predictive check (VPC), parameter precision, and objective function values (OFVs), such as the negative twice log likelihood (−2LL) and the Bayesian Information Criteria (BIC).

Compartment models with extravascular administration and first‐order elimination were fitted to the CBD/CBDA concentration‐time data. All parameters were assumed to be log‐normally distributed. A multiplicative (proportional) residual error model was applied. Observations below the lower limit of quantification (LOQ) were censored, allowing the model to predict any concentration between zero and LOQ (M4 method). Age, bodyweight, and sex were evaluated as covariates. To qualify for inclusion in the final model, a covariate should lead to a reduction in both between‐subject variability (BSV) and the objective function value (OFV). Additionally, parameter precision should improve, the correlation between the covariate and the parameter should be statistically significant (as assessed by Pearson's correlation), and the covariate estimate should be significantly different from zero (as determined by Wald's test).

Between subject variability (BSV) was modeled as:

(1) $$\theta_{i}=\theta_{\mathrm{tv}}\cdot \exp\left(\eta_{i}\right)$$

where *θ*_*tv*_ is the typical population value of the parameter, *θ*_*i*_ is the value of the pharmacokinetic parameters in the *i*th horse, and *η*_*i*_ is the deviation from the corresponding population value associated to the *i*th horse.

The estimated standard deviation of the random effects (*ω*), as reported by Monolix, was converted to a coefficient of variation (CV%) using Equation (2) and used to describe interindividual variability (IIV):

(2) $$\mathrm{CV}\%=\sqrt{\exp\left(\omega^{2}\right)-1}\cdot 100$$

Shrinkage of the random effects (eta) toward the population mean was calculated as:

(3) $$\text{shrinkage}=1-\frac{\mathrm{SD}\left(\eta_{r}\right)}{\omega }$$

where SD(*η*_*r*_) is the empirical standard deviation of the random effects. When shrinkage for eta was > 51%, the random component was not considered robustly estimated.

### 2.7 Statistical Analyses of Hematological and Biochemical Variables

To assess the normality of the data distribution, quantile–quantile (QQ) plots were examined. For each variable, the change from baseline (pre‐ to post‐treatment) was calculated within each of the three treatment groups. These change scores served as the dependent variable in a linear mixed‐effects model, which included treatment as a fixed effects and subject as a random effects.

For variables with values below the LOQ, 50% of the LOQ was imputed. All statistical analyses were performed using R version 4.3.0. Residual plots were visually inspected to evaluate the model assumptions regarding variance.

Statistical significance between treatment groups was assessed using the *F*‐test based on type III sum of squares, with degrees of freedom determined by the Kenward–Roger method. Post hoc comparisons between each active treatment and placebo groups were performed using Dunnett's test.

### 2.8 Artificial Intelligence Disclosure

No artificial intelligence (AI) technology was used to develop any portion of the manuscript.

## 3 Results

### 3.1 CBD and CBDA in Plasma

**FIGURE 1:**
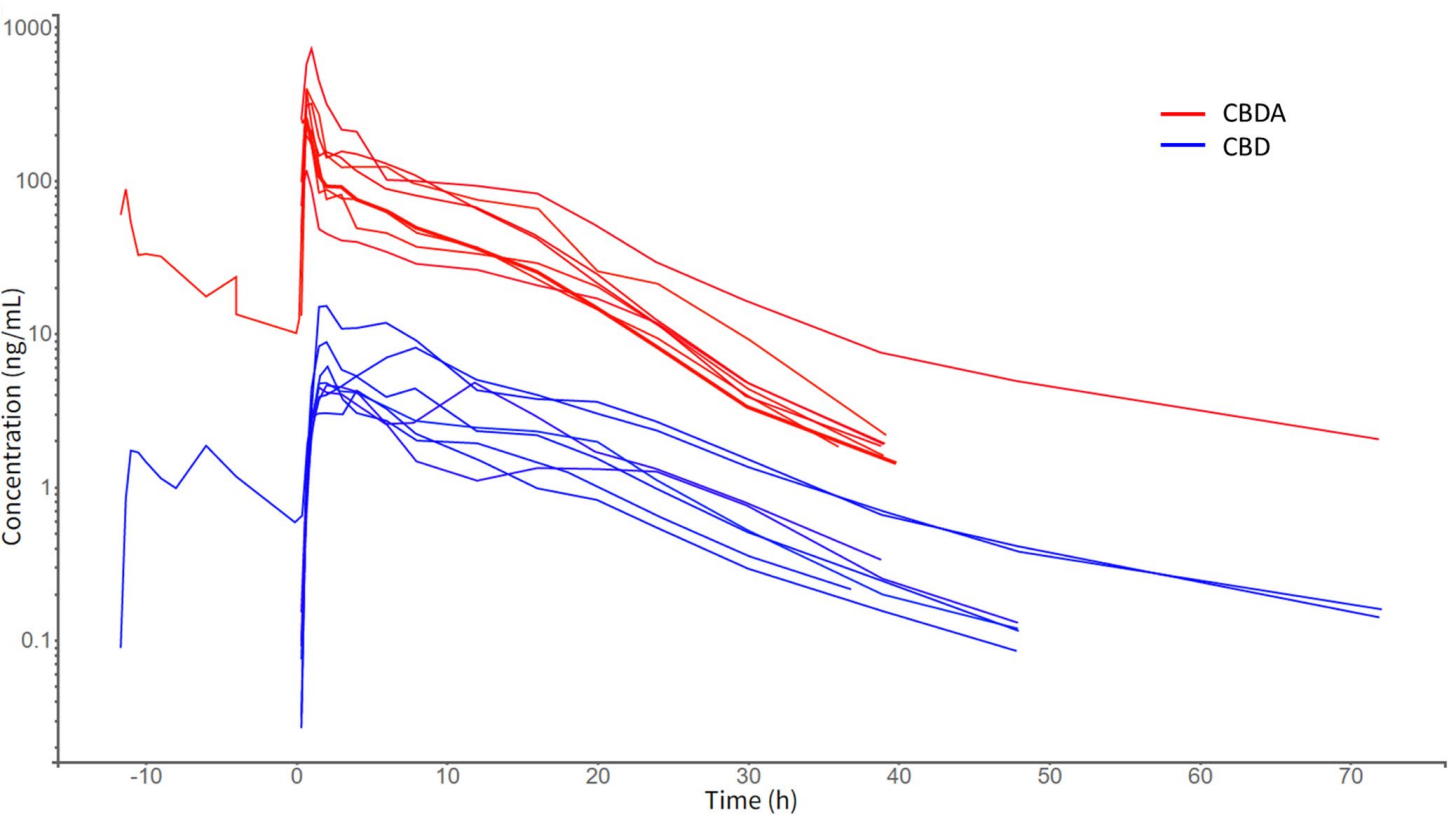
Spaghetti plot showing plasma concentration‐time courses following oral administration of 3 mg/kg cannabidiol (CBD, blue) and cannabidiolic acid (CBDA, red) to eight horses. One horse was administered 1 mg/kg followed by 2 mg/kg after 12 h, explaining measured concentrations at time points before 0 h.

The plasma concentration‐time profile of CBD was characterized by a rapid absorption phase, with peak concentrations observed between 1.5 and 4 h post‐administration. For CBD, median (range) maximum observed plasma concentration (*C*_max_) and AUC_0–ꚙ_ were 5.04 ng/mL (4.26–15.2 ng/mL) and 74.82 ng h/mL (45.50–186.93 ng h/mL), respectively. The CBD plasma concentrations declined with a median (range) terminal half‐life (*t*_*1/2z*_) of 7.8 h (6.9–15.0 h). The CBD levels were below the LOQ by 52 h in 6 horses and by 96 h in all horses (Figure 1).

In contrast, CBDA plasma concentrations rose more rapidly than those of CBD, with peak concentrations occurring between 0.67 and 1 h. Median (range) CBDA plasma *C*_max_ and AUC_0–ꚙ_ were 283.5 ng/mL (117.0–725.0 ng/mL) and 1518.96 ng h/mL (805.44–3495.88 ng h/mL), respectively. Following an initial rapid decline in plasma concentrations, a slower terminal phase was observed, with a median (range) *t*_*1/2z*_ of 5.3 h (4.6–17.9 h). Individual parameter values from the non‐compartment analyses of CBD and CBDA in plasma are shown in Table 1. The median (range) CBDA/CBD ratio for *C*_max_ and AUC_0–ꚙ_ was 47.5 (25–67) and 17.5 (14‐36), respectively.

**TABLE 1:** Results for the non‐compartmental analyses of plasma data following oral administration of 3 mg/kg cannabidiol (CBD) and 3 mg/kg cannabidiolic acid (CBDA) in eight horses in a crossover study.

Horse	*C*_max_ (ng/mL)		AUC_0–ꚙ_ (ng h/mL)		*k* (1/h)		*t*_*1/2z*_ (h)	
	CBD	CBDA	CBD	CBDA	CBD	CBDA	CBD	CBDA
1^a^	6.11	208.5	55.37	1121.18	0.091	0.14	7.6	5.1
2	8.83	400.0	152.34	2226.53	0.063	0.15	11.1	4.6
3	5.28	249.0	77.24	1267.42	0.082	0.11	8.4	6.1
4	4.26	226.0	48.50	1770.50	0.086	0.14	8.0	5.1
5	4.80	389.0	84.20	1190.94	0.092	0.13	7.5	5.3
6	4.78	318.0	72.39	2036.46	0.10	0.13	6.9	5.2
7	4.61	117.0	58.12	805.44	0.098	0.12	7.1	5.9
8	15.20	725.0	186.93	3495.88	0.046	0.039	15.0	17.9
Median	5.04	283.5	74.82	1518.96	0.089	0.13	7.8	5.3

*Note:* *C*_max_, AUC_0–ꚙ_, *k* and *t*_*1/2z*_ are the maximum observed concentration, the area under the curve from time 0 to eternity, the elimination rate constant and the terminal half‐life, respectively.

^a^ Horse #1 was administered 1 mg/kg followed by 2 mg/kg after 12 h.

A one‐compartment model with lag time, first‐order absorption, and elimination provided the best fit of the CBD data. Parameters reported for this model were the absorption rate constant (*k*_*a*_), apparent volume of distribution relative to the bioavailability (*V/F*), and the clearance relative to the bioavailability (Table 2). For CBDA data, a two‐compartment model based on clearance and volume parameterization (relative to the bioavailability) with lag time provided the best fit. None of the covariates—age, bodyweight, or sex—significantly improved the model fit for either CBD or CBDA and were therefore not included in the final models.

**TABLE 2:** Non‐linear mixed effects model parameter estimates after oral administration of 3 mg/kg cannabidiol (CBD) and 3 mg/kg cannabidiolic acid (CBDA) to eight horses in a crossover study.

	Unit	Typical value	RSE	IIV (%)
Model parameters CBD				
*k*_*a*_	1/h	1.32	19.7	41.72
*t*_*lag*_	h	0.45	10.3	24.2
*Cl/F*	L/h/kg	35.41	15.7	44.79
*V/F*	L/kg	454	12.3	33.26
Residual error parameter		0.25	7.47	—
Model parameters CBDA				
*k*_*a*_	1/h	3.51	15.4	8.284
*t*_*lag*_	h	0.28	7.67	13.04
*Cl/F*	L/h/kg	1.92	15.6	44.66
*V1/F*	L/kg	3.19	35.0	6.43
*V2/F*	L/kg	13.53	21.1	58.13
*Q*	L/h/kg	11.67	42.2	115.68
Residual error parameter		0.22	7.54	—

*Note:* *k*_*a*_, *t*_*lag*_, *Cl/F*, *V/F V1/F*, *V2*/*F* and *Q* are the absorption rate constant, the lag‐time, the ratio of the clearance and the bioavailability, the ratio of the volume and the bioavailability, the ratio of the volume of the central compartment and the bioavailability, the ratio of the volume of the peripheral compartment and the bioavailability and the intercompartmental clearance, respectively. RSE is the relative standard error of the typical value and IIV (%) is the inter individual variability.

**FIGURE 2:**
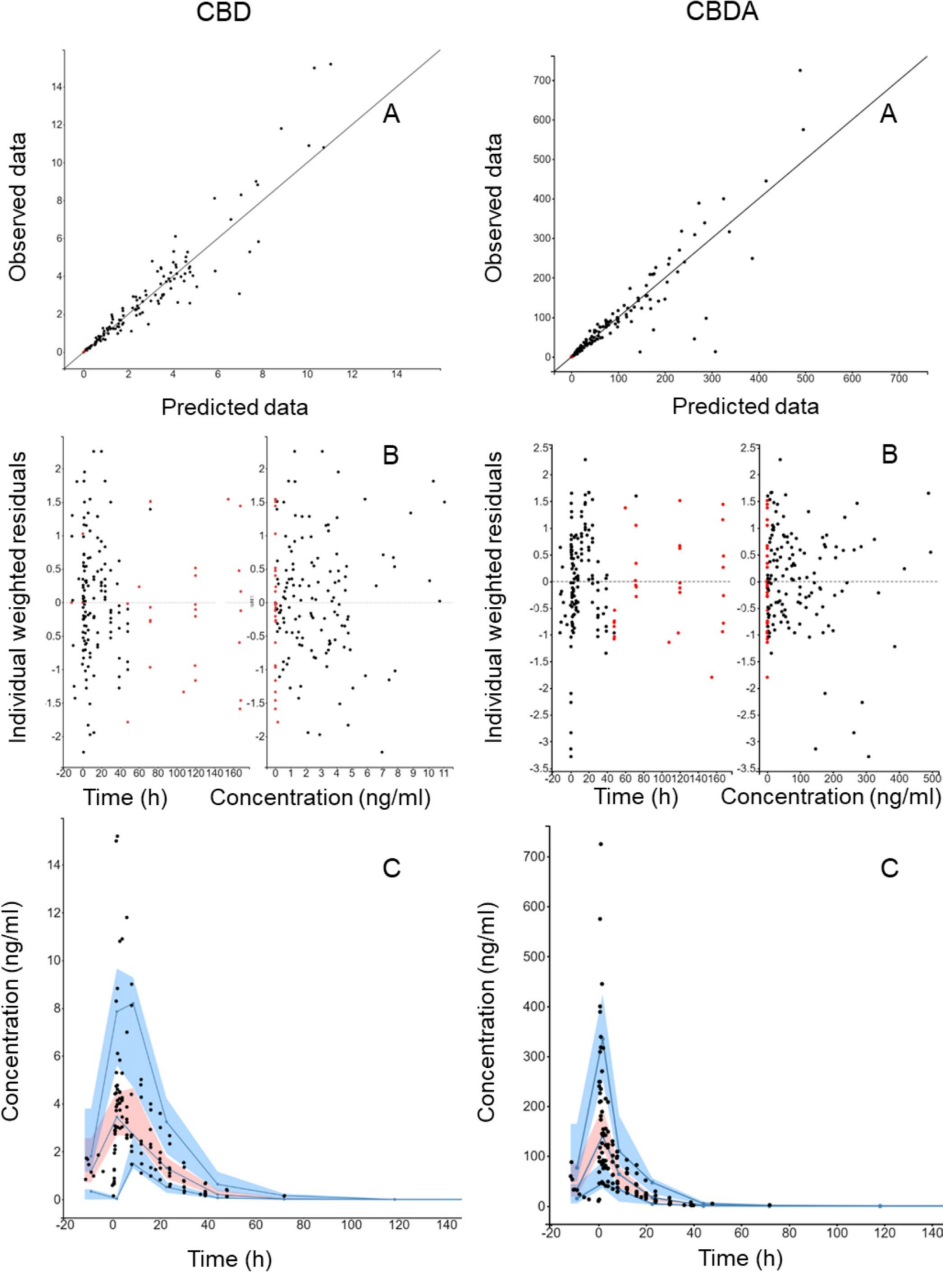
Goodness of fits plots for the pharmacokinetic model. Cannabidiol (CBD) data is shown in the left column and cannabidiolic acid (CBDA) data is shown in the right column. (A) Observed individual concentrations VS predicted individual concentrations plot, (B) Individual weighted residuals VS. time and concentration and (C) Visual predictive check. Filled black circles represent observed data and filled red circles represent model predicted concentrations below quantification limit. The solid line in plot A represent the line of unity (observation = prediction). The solid lines in plot C represent the 10th, 50th and 90th empirical percentile, respectively. The blue shaded areas in plot C is the 10th and the 90th prediction interval and the red shaded area is the median prediction interval.

The final models adequately described the experimental data (Figure 2). Parameter estimates and their associated precision are shown in Table 2. Shrinkage values were below 10% for all parameters, indicating robust estimation. In total, 23 samples (15% individual range: 2–3 or 10.5%–16% per horse) were censored in CBD analyses. For CBDA, 27 samples (17.5% individual range: 2–4 or 10.5%–21% per horse) were censored.

### 3.2 CBD and CBDA in Urine

Maximum urine concentrations of CBD (median 378 ng/mL, range 79.6–4490 ng/mL) and CBDA (median 2505 ng/mL, range 1585–3990 ng/mL) were observed in samples collected 4 h or 12 h post‐administration. The median (range) CBD and CBDA AUC_0–ꚙ_ were 6719 ng h/mL (2339–60,214 ng h/mL) and 35,333 ng h/mL (20,736–62,829 ng h/mL), respectively. The CBD concentrations in urine remained above the LOQ (0.5 ng/mL) throughout the 168 h sampling period in 3 horses. In contrast, CBDA concentrations in urine fell below the LOQ (5 ng/mL) by 120 h post‐administration. The median (range) CBD and CBDA terminal half‐lives in urine were 15.5 (5.9–86 h) and 6.9 h (4.4–10.3 h), respectively. Individual parameter values from the non‐compartment analyses of CBD and CBDA in urine can be seen in Table 3. The median (range) CBDA/CBD ratio in urine was 7 (0.7–30) and 5 (0.9–10) for *C*_max_ and AUC_0–ꚙ_, respectively.

**TABLE 3:** Results for the non‐compartmental analyses of urine data following oral administration of 3 mg/kg cannabidiol (CBD) and 3 mg/kg cannabidiolic acid (CBDA) to eight horses in a crossover study.

Horse	*C*_max_ (ng/mL)		AUC_0–ꚙ_ (ng h/mL)		*k* (1/h)		*t*_*1/2z*_ (h)	
	CBD	CBDA	CBD	CBDA	CBD	CBDA	CBD	CBDA
1^a^	329	2660	7043	62,829	0.12	0.11	5.9	6.6
2	4490	3410	60,214	52,877	0.015	0.16	47.0	4.4
3	712	2125	10,291	31,312	0.048	0.096	14.5	7.2
4	418	3910	5607	58,028	0.042	0.14	16.5	4.9
5	338	2865	6547	36,607	0.078	0.086	8.8	8.1
6	79.6	2350	2339	30,242	0.077	0.11	9.0	6.2
7	273	1585	5002	20,736	0.0081	0.093	86.0	7.4
8	487	2025	10,455	34,058	0.0025	0.067	28.2	10.3
Median	378	2505	6719	35,333	0.045	0.103	15.5	6.9

*Note:* *C*_max_, AUC_0–ꚙ_, *k*, and *t*_*1/2z*_ are the maximum observed concentration, the area under the curve from time 0 to eternity, the elimination rate constant and the terminal half‐life, respectively.

^a^ Horse #1 was administered 1 mg/kg followed by 2 mg/kg after 12 h.

**FIGURE 3:**
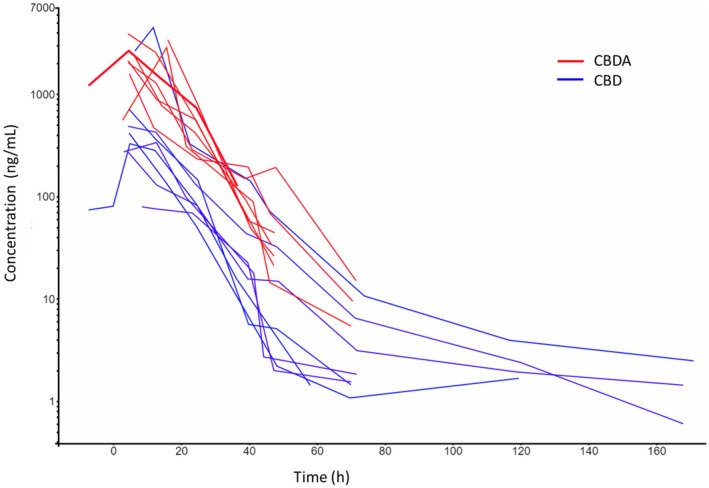
Spaghetti plot showing urine concentration‐time courses following oral administration of 3 mg/kg cannabidiol (CBD, blue) and cannabidiolic acid (CBDA, red) to eight horses. One horse was administered 1 mg/kg followed by 2 mg/kg after 12 h, explaining measured concentrations at time points before 0 h.

The urine concentration‐time profiles are shown in Figure 3.

### 3.3 Hematology and Biochemistry

No significant differences were observed in any biochemical or hematological variables when comparing the CBD or CBDA treatments to the control treatment.

## 4 Discussion

The primary objective of this study was to investigate and compare the plasma exposure of CBD and CBDA following oral administration of equivalent doses to horses. Before any drug is introduced for clinical use in animals, its pharmacokinetics, pharmacodynamics, and safety profile should be characterized. In the absence of such data—particularly regarding plasma exposure as it relates to effective concentrations or doses—there is a risk of administering suboptimal treatments that could compromise animal welfare.

The main finding of this study is the marked difference in plasma exposure between CBD and CBDA, consistent with previous observations in horses receiving combined CBD and CBDA (Thomson et al. 2024). A similar difference was observed in urine, although it was less pronounced due to one outlier horse which had 5–10 times higher urinary CBD concentrations than others. The molecular weight of CBDA (358.47 g/mol) is slightly higher than that of CBD (314.47 g/mol), corresponding to a ratio of 1.14. On a molar basis, the administered doses were comparable (9.54 μmol/kg for CBD and 8.37 μmol/kg for CBDA), yet the observed plasma exposure ratio (CBDA AUC_0–∞_/CBD AUC_0–∞_) was 17.5 (range 14–36). This large difference in exposure cannot be explained by the small difference in molecular weight or molar dose, suggesting that CBDA is either absorbed more efficiently from the gastrointestinal tract or undergoes less first‐pass metabolism and clearance than CBD.

Oral bioavailability of CBD in horses has previously been reported to be low, ranging from 8% to 14% (Sánchez de Medina et al. 2023; Turner et al. 2022). Gastrointestinal (GI) absorption depends on several factors (Alqahtani et al. 2021), including stability and solubility of the compound in the GI tract, mucosal permeability, and affinity to transport (influx or efflux) proteins. Feeding state (fed vs. fasted) may also influence absorption, since certain drug molecules, such as phenylbutazone in horses, can bind to digesta or undergo co‐transport (Lees et al. 1988). Additionally, pharmaceutical formulation can affect systemic exposure after extravascular administration (Gupta et al. 2013). Relevant for comparison with this study, Sánchez de Medina et al. (2023) investigated two different oral CBD formulations and modeled the absorption using a time‐dependent (Weibull) function. Compared with the present study, those two oral formulations exhibited slower absorption (later time for *C*_max_ with a less sharp peak in the curve), for which a Weibull function was appropriate. In our study, absorption was rapid, the profiles were well described by simple first‐order input, and a Weibull term was unnecessary—hence the larger *k*_*a*_ estimate (rapid input relative to elimination). The plasma concentration‐time profile of CBD in this study also aligns with previously reported data using the same or similar dosing regimens in horses (Turner et al. 2022; Ryan et al. 2021; Yocom et al. 2022; Eichler et al. 2023), including a comparable terminal half‐life in plasma. In contrast, in another study, higher doses of CBD were modeled using a three‐compartment model (compared to a one‐compartment model in this study) with a markedly longer terminal half‐life than in the present study (> 24 h vs. 8 h) (Sánchez de Medina et al. 2023). One reason for the use of a three‐compartment model in that study was probably that one dose was given intravenously. Using only oral administration as in this study, the initial distribution phase cannot be identified as it can after intravenous administration. However, that does not explain the difference in terminal half‐life. The present study sampled more frequently and for a longer time after administration compared with the previous. Moreover, the LOQ of the analytical method was lower in this study. This should have allowed identification of the terminal phase of the plasma concentration‐time course. Thus, the difference is most likely caused by other factors, such as methodology. In this context, it is almost mandatory to mention that the model is fitted to the data set, not vice versa. Consequently, different models may provide different representations of data. Hence, the terminal half‐life of a one‐compartment model reflects both distribution to and elimination from both highly and poorly perfused peripheral compartments. Therefore, it is not directly comparable to the terminal half‐life of a three‐compartment model that reflects elimination from the poorly perfused compartment. Moreover, it can be speculated that the higher dose given by Sánchez de Medina et al. (2023) caused a larger concentration gradient between plasma and the peripheral tissues. In turn, that could cause increased uptake into and elimination from the poorly perfused compartments, which was accurately captured by the three‐compartment model and reflected in a longer terminal half‐life. Further on, repeated dosing has been shown to result in accumulation of CBD and a prolonged terminal half‐life of approximately 161.29 ± 43.65 h (Eichler et al. 2023), indicating distribution to peripheral compartments. This is supported by the relatively large steady‐state volume of distribution (~36 L/kg) described by Sánchez de Medina et al. (2023). Such accumulation, combined with a potentially high potency, could explain case reports of positive effects on stereotypic behaviors and allodynia in horses treated with daily doses of 0.5 mg/kg (Ellis and Contino 2021; Zamith Cunha et al. 2023). At slightly lower doses, CBD has been reported to lack sedative, ataxic, or cardiac effects in horses (St Blanc et al. 2022; Draeger et al. 2021). Together with hematology and biochemistry data from this and previous studies (Yocom et al. 2022), this suggests that short‐term treatment with low to moderate doses (≤ 0.5 mg/kg orally) is likely to be well tolerated, with minimal or mild side effects. However, future studies demonstrating its effect on other clinical outcomes, for example, the anti‐inflammatory or analgesic effects, using higher doses and longer treatment durations, are warranted to evaluate the clinical potential of CBD in horses.

The CBDA peak plasma concentrations were 25–67‐fold higher than those of CBD despite equivalent dosing, which is consistent with findings from a recent multidose study (Wang et al. 2025). This difference was also reflected in the total plasma exposure (AUC_0–ꚙ_), which was 14–36‐fold larger after CBDA administration than after CBD administration. However, since neither that study nor the current one included intravenous administration, absolute bioavailability could not be calculated. Nevertheless, the substantially larger area under the plasma concentration‐time curve (AUC) for CBDA strongly suggests greater bioavailability, possibly due to improved absorption or reduced first‐pass metabolism and/or clearance. The current study design does not allow determination of the exact mechanism.

Higher plasma exposure increases the likelihood of the compound exceeding the potency threshold and achieving therapeutic concentrations. CBDA has been suggested to be more potent than CBD in rodent models (Rock et al. 2018), and therefore may offer superior clinical efficacy. However, CBDA has been shown in the literature to be less chemically stable than CBD, but recently published studies have raised a promising alternative to CBDA, CBDA‐methyl ester. This compound is a semi‐synthetic analog with improved stability, which has efficacy in rodent models of pain and nausea (Zhu et al. 2020; Pertwee et al. 2018). Considering the current findings, a logical next step would be to conduct a full pharmacokinetic study of CBDA‐methyl ester in horses, followed by pharmacodynamic assessments and clinical trials to evaluate its therapeutic potential.

## Author Contributions

C.E., M.S., and E.H. planned the study. C.E., P.M., M.S., K.S., and E.H. performed the experiment and plasma analyses. C.E., P.M., and R.G. analysed the data. P.M. and C.E. drafted the manuscript with input from M.S. and K.S. All authors revised the manuscript and approved the final version of the manuscript.

## Ethics Statement

The study protocol was approved by the Animal Ethics Committee, Uppsala, Sweden (5.8.18‐02809/2020).

## Conflicts of Interest

The authors declare no conflicts of interest.

## Data Availability

The data that support the findings of this study are available from the corresponding author upon reasonable request.
